# Crossing boundaries in neurosurgical education: The Pan-African EANS-supported course as a blueprint for global capacity building

**DOI:** 10.1016/j.bas.2026.105930

**Published:** 2026-01-06

**Authors:** Ondra Petr, Christian Preuss-Hernández, Nicephorus B. Rutabasibwa, Marta Garvayo, Romani Sabas, Andreas K. Demetriades, Magnus Tisell, EANS Global & Humanitarian Committee

**Affiliations:** a Department of Neurosurgery, Muhimbili Orthopaedic & Neurosurgery Institute, Dar es Salaam, Tanzania; b Department of Neurosurgery, Medical University Innsbruck, Innsbruck, Austria; c Department of Neurosurgery, Groupe Hospitalier Universitaire (GHU) Paris Psychiatrie & Neurosciences, Paris, France; d Department of Neurosurgery, Royal Infirmary, Edinburgh, UK; e Department of Neurosurgery, Sahlgrenska University Hospital, Göteborg, Sweden; f Global and Humanitarian Neurosurgery Committee, European Association of Neurosurgical Societies (EANS), Brussels, Belgium

**Keywords:** Neurosurgical education, Global neurosurgery, Capacity building, Africa, Low- and middle-income countries (LMIC), International collaboration

## Abstract

**Introduction:**

Neurosurgical training in Africa is critically limited and expensive. The European Association of Neurosurgical Societies(***EANS***) Global Humanitarian Committee partnered with Continental African Neurosurgical Societies(***CAANS***), West African College of Surgeons(***WACS***), and College of Surgeons of East, Central & Southern Africa(***COSECSA***) to adapt its established curriculum of the training courses for African residents&early-career neurosurgeons and piloted the First Pan-African course in May 2025.

**Research question:**

Does a collaborative, context-adapted international Pan-African EANS course enhance neurosurgical knowledge in resource-limited African settings and prove feasible for capacity building in LMIC practice?

**Materials and methods:**

The five-day Pan-African Neurosurgery Training Course (May 2025, Dar es Salaam, Tanzania) covered vascular *neurosurgery&skull base*. Fifty-eight pre-course and 61 post-course surveys assessed participant demographics, institutional resources, baseline/post-training self-rated knowledge (5-point scale), and feedback from 19 nations.

**Results:**

Mean knowledge scores increased from 2.5 → 4.1 for vascular neurosurgery (64.0 % improvement, P < 0.001) and 2.6 → 4.0 for skull base (53.8 % improvement, P < 0.001). Overall course quality was 4.7/5. Interactive formats (breakout sessions/discussion groups) were highest-rated (4.8/5), content adaptation to LMIC-settings was 4.5/5. All participants expressed interest in future courses; 95.1 % were willing to serve as future faculty and indicated institutions could host future courses. Interest in partnerships: training workshops(90.2 %), research collaboration(90.2 %), fellow exchanges(88.3 %).

**Discussion and conclusion:**

This inaugural Pan-African EANS-supported training course demonstrates that collaborative, contextually adapted education effectively enhances neurosurgical knowledge in resource-constrained settings. Exceptional satisfaction, substantial knowledge gains, and universal demand for continuation provide strong evidence for program expansion. This reproducible model establishes a scalable framework for sustainable capacity-building across Africa.

## Highlights

• First Pan-African neurosurgical training course for 97 participants from 19 nations.
• Knowledge gains of 53.8 % (skull base) and 64.0 % (vascular neurosurgery) achieved.
• Interactive formats rated highest; course adapted for low-resource settings.
• 100 % of participants desire continuation; 95 % willing to serve as future faculty.
• Scalable collaborative model demonstrated for sustainable capacity building.

## 1 Introduction

Since the 1970s, the European Association of Neurosurgical Societies (EANS) has developed a comprehensive four-year training curriculum for neurosurgery residents across Europe, systematically covering all major subspecialties including Vascular Neurosurgery, Peripheral Nerves, Tumors, Functional Neurosurgery, Head Injury and Spine. These courses, conducted as intensive four-to five-day sessions, have become highly valued educational experiences for European trainees, combining didactic lectures, interactive discussion groups, and breakout sessions that ensure high participant-to-faculty ratios.

The global burden of neurosurgical disease disproportionately affects low- and middle-income countries (LMICs), particularly in Africa, where access to specialized neurosurgical care remains severely limited (Cadieux et al., 2025; Park et al., 2016). Currently, only 32 of Africa's 54 nations offer neurosurgical residency programs (Waterkeyn et al., 2023), and the continent faces a critical workforce shortage with approximately 0.15 neurosurgeons per million population – far below the World Health Organization's recommended ratio of 1 per 200,000 (Haglund et al., 2017). This disparity necessitates a 1700 % increase in the neurosurgical workforce to meet current demand (Haglund et al., 2017).

Training opportunities for African neurosurgeons are scarce and often financially prohibitive, particularly for residents who typically receive minimal or no income during their training years (Cadieux et al., 2025; Park et al., 2016). International fellowship opportunities, while valuable, are frequently inaccessible due to cost barriers and limited availability. Despite these challenges, supplemental training initiatives have demonstrated effectiveness in enhancing neurosurgical knowledge, clinical confidence, and technical competency in LMIC settings (Waterkeyn et al., 2023).

Following extensive consultations between the EANS Global Humanitarian Committee (GHC) and key African neurosurgical organizations – including the Continental Association of African Neurosurgical Societies (CAANS), the College of Surgeons of East, Central and Southern Africa (COSECSA), and the West African College of Surgeons (WACS) – a collaborative decision was reached to adapt the successful EANS training model for the African context. This initiative represents the first Pan-African training course in neurosurgery specifically designed and implemented for the entire continent, addressing a critical gap in specialized neurosurgical education within LMIC regions.

This paper describes the comprehensive organization, execution, challenges encountered, and outcomes of this inaugural educational initiative, including detailed evaluation data from participating trainees. The findings provide insights into the feasibility and effectiveness of international collaborative educational models in resource-limited settings.

## 2 Material and methods

### 2.1 Course organization and structure

The inaugural African Neurosurgical Training Course was conducted over five days in May 2025 at the Muhimbili Orthopaedic & Neurosurgery Institute (MOI) in Dar es Salaam, Tanzania. The course focused on two complex subspecialties: vascular neurosurgery and skull base. Organization was led by the EANS GHC in close collaboration with the local Tanzanian neurosurgery team, under the leadership and endorsement of CAANS, COSECSA and WACS. Course directors included Nicephorus B. Rutabasibwa (Tanzania), Magnus Tisell (EANS GHC), and Ondra Petr (EANS GHC).

### 2.2 Curriculum development

The curriculum was jointly developed by the GHC and African Organizing (Task Force) Committees, drawing upon the established EANS training course framework while incorporating specific adaptations for LMIC contexts. The program featured internationally recognized neurosurgical faculty and employed a multimodal teaching approach combining plenary lectures, small-group breakout sessions, and interactive discussion groups designed to maximize participant engagement. To enhance preparation, multiple pre-course lectures were delivered via videoconference in the months preceding the in-person course.

### 2.3 Faculty composition

A total of 22 faculty members participated, including 12 from European countries, 6 from other African nations, and 4 local faculty from Tanzania. This international composition ensured both technical expertise and contextual relevance to African practice environments.

## 3 Funding and scholarships

Total funding of €37,400 was secured through collaborative fundraising efforts led by the EANS GHC. Major contributors included the Olympus Foundation (€16,000), the EANS Foundation (€10,000), and the British Society of Neurologic Surgeons – the British Fund Diana and Alan Karter Global Neurosurgery (£10,000). Participation was open to all African neurosurgeons and trainees. Twenty-five merit-based scholarships were awarded to cover travel expenses, course registration fees, and accommodation based on curriculum vitae evaluation, with an additional two participants receiving institutional sponsorship.

### 3.1 Data collection and analysis

To comprehensively evaluate the course and assess participant needs, pre- and post-course surveys were administered electronically via Google Forms, distributed through QR codes. The pre-course survey collected demographic data, training level, institutional resources and equipment availability, prior surgical experience, and baseline self-rated knowledge in vascular and skull base neurosurgery. For the purposes of the survey, “skull base surgery” was defined as cranial procedures involving lesions located at or adjacent to the anterior, middle, or posterior cranial fossa floor, including pituitary, olfactory groove, sphenoid wing, petroclival, and cerebellopontine angle lesions, regardless of whether highly specialized skull base approaches or advanced technological adjuncts were available at the institution. The post-course survey reassessed knowledge levels, evaluated quality ratings for various educational formats, gathered feedback on program satisfaction, identified preferred topics for future courses, and assessed interest in international collaborative partnerships. Self-rated knowledge was assessed using a five-point Likert scale (1 = minimal knowledge, 5 = expert knowledge).

## 4 Results

### 4.1 Participant demographics and geographic representation

**Fig. 1:**
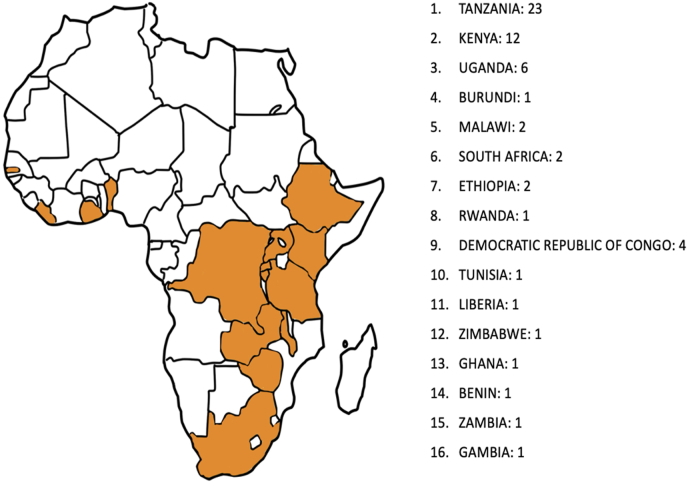
**Geographic Distribution of Course Participants** Map of Africa highlighting the 16 countries represented by participants who completed the pre-course survey (displayed in orange). Countries represented include participants from East Africa (Tanzania, Kenya, Uganda, Rwanda, Ethiopia), West Africa (Nigeria, Ghana, Senegal, Cameroon), Central Africa (Democratic Republic of Congo), and Southern Africa (South Africa, Zimbabwe, Zambia, Malawi, Botswana, Mozambique), demonstrating the Pan-African scope of the inaugural course.

Of 97 total course attendees from 19 African countries, 58 participants (59.8 %) completed the pre-course survey and 61 participants (62.9 %) completed the post-course survey, representing 16 different African nations (Fig. 1). Geographic representation included participants from East, West, Central, and Southern African regions, demonstrating the Pan-African reach of the initiative. Twenty-five participants (43.1 %) received EANS-funded scholarships, with two additional participants (3.4 %) supported through other institutional sources.

Training level distribution among pre-course respondents included 31 neurosurgery residents (53.4 %), 19 neurosurgery specialists (32.8 %), 7 registrars (12.1 %), and 1 medical student (1.7 %). This diversity in training stages facilitated peer learning and knowledge exchange across different experience levels.

### 4.2 Institutional resources and infrastructure

**Fig. 2:**
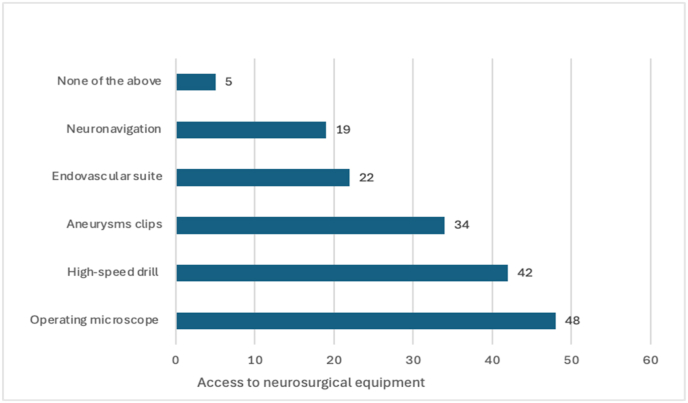
Institutional access to essential neurosurgical equipment.

Assessment of institutional capacity revealed significant variability in access to essential neurosurgical equipment across participating centers (Fig. 2). Among 58 pre-course respondents, 48 (82.8 %) reported institutional access to operating microscopes, 42 (72.4 %) to high-speed drills, 34 (58.6 %) to aneurysm clips, 22 (37.9 %) to endovascular suites, and 19 (32.8 %) to neuronavigation systems. Notably, 5 participants (8.6 %) reported having access to none of the listed equipment, highlighting severe resource constraints in some centers.

Most participants practiced at institutions where vascular (N = 35, 60.3 %) and skull base (N = 50, 86.2 %) neurosurgical procedures are performed. However, only 17 institutions (29.3 %) maintained dedicated specialized sections for these complex subspecialties, suggesting that while basic procedures may be attempted, dedicated expertise and infrastructure for advanced cases remain limited.

### 4.3 Pre-course knowledge assessment

Baseline self-rated knowledge scores on the five-point scale revealed significant knowledge gaps in both subspecialties: skull base surgery mean score 2.6 (95 % CI: 2.4–2.8), vascular neurosurgery mean score 2.5 (95 % CI: 2.3–2.7). These scores, falling between “basic knowledge” and “moderate knowledge,” indicated substantial opportunity for educational intervention.

### 4.4 Post-course evaluation and knowledge gains

**Fig. 3:**
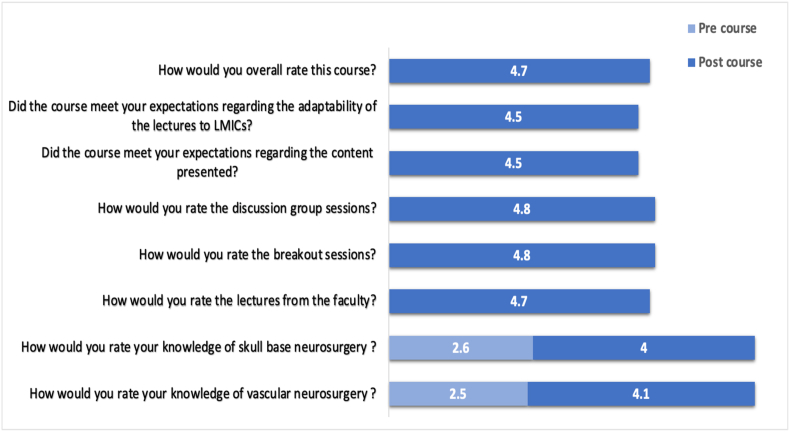
**Course Quality Ratings and Knowledge Improvement** Participant evaluation of course quality and self-rated knowledge gains. All ratings are on a five-point Likert scale. Knowledge in skull base and vascular neurosurgery was assessed both pre- and post-course, demonstrating substantial improvements: skull base surgery increased from 2.6 to 4.0 (53.8 % improvement), and vascular neurosurgery increased from 2.5 to 4.1 (64.0 % improvement). Overall course quality was rated 4.7/5, with interactive formats (discussion groups and breakout sessions) receiving the highest ratings at 4.8/5. Course content relevance and LMIC adaptation were both rated 4.5/5.

Among 61 post-course respondents, the course received exceptional overall quality ratings of 4.7 out of 5 (Fig. 3). Participants reported that the course effectively met their expectations regarding content relevance (4.5/5) and appropriate adaptation of lectures to LMIC practice settings (4.5/5).

Substantial and statistically significant improvements in self-reported knowledge were documented. Vascular neurosurgery knowledge scores increased from 2.5 to 4.1, representing a 64.0 % improvement (P < 0.001). Mean skull base surgery knowledge scores increased from 2.6 pre-course to 4.0 post-course, representing a 53.8 % improvement (P < 0.001). These scores, approaching “good knowledge” to “advanced knowledge” levels, demonstrate meaningful educational impact.

### 4.5 Educational format effectiveness

Different educational modalities received varying satisfaction ratings. Interactive formats achieved the highest ratings: discussion groups (4.8/5) and breakout sessions (4.8/5) were most valued by participants, reflecting strong preference for active learning over passive didactic methods. Traditional plenary lectures received solid ratings (4.6/5), while pre-recorded online content was rated lower (4.3/5). This feedback pattern underscores the importance of in-person, interactive educational experiences in this context.

### 4.6 Qualitative feedback analysis

Systematic analysis of open-ended feedback identified key strengths and areas for improvement (Table 1). Participants most valued the interactive teaching formats, particularly breakout sessions, discussion groups, and case-based learning. Skull base surgery sessions were specifically highlighted as valuable given the limited exposure to this subspecialty in many African training programs.

**Table 1:** **Summary of Participant Feedback on Program Strengths and Areas for Improvement** Qualitative analysis of open-ended participant feedback identified key program strengths and specific recommendations for future course iterations.

If you had to change one thing in the program presented this week, what would that be?	If you had to keep one session in the program presented this week, what would that be?
More hands-on sessions (cadaveric dissections, live surgeries, simulations, dummy training)	Breakout sessions (most frequently mentioned, highly valued)
Increase skull base surgery focus (more lectures, practical sessions, simulations)	Discussion groups (group learning highly appreciated)
Better time management & scheduling (start on time, adjust duration, extend course length)	Skull base surgery sessions (widely regarded as valuable)
Reduce reliance on online/pre-recorded lectures (seen as less useful than in-person)	Lectures (especially stroke, aneurysms, tumors)
Add more practical surgical scenarios & case discussions	Case discussions (seen as very effective for learning)
Improve logistics (venue, sound systems, course timing, location convenience)	Vascular/stroke management sessions (key clinical areas valued)
Introduce new teaching formats (live theater sessions, virtual reality, one-on-one sessions)	Combined teaching formats (discussion + breakout + lectures mix)

The most frequently cited suggestion for improvement was incorporation of additional hands-on training opportunities, including cadaveric dissections, live surgical demonstrations, and simulation-based learning. Participants also requested expanded skull base surgery content, improved time management and scheduling, and reduced reliance on pre-recorded lectures in favor of live instruction. These recommendations provide clear direction for future course iterations.

### 4.7 Interest in future courses and partnerships

Enthusiasm for program continuation was universal, with all 61 respondents (100 %) expressing interest in participating in future course cycles. Remarkably, 58 participants (95.1 %) indicated willingness to serve as faculty in future courses, and an equal number (95.1 %) indicated their home institutions could potentially host future courses, suggesting strong commitment to sustainable capacity building.

For the 2026 course cycle, the most requested topics were pediatric neurosurgery and neuro-oncology (N = 36, 59.0 %), followed by spine surgery (N = 32, 52.5 %), reflecting perceived training gaps in these areas.

### 4.8 Collaborative partnership interest

**Fig. 4:**
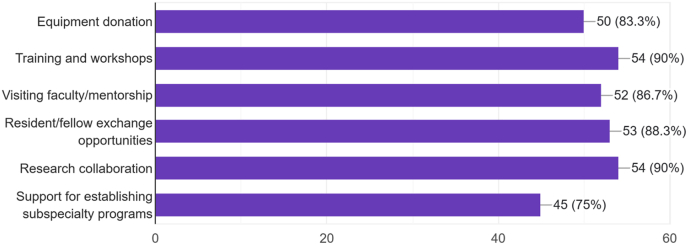
**Desired Types of International Collaboration and Support** Types of support or collaboration that participants' institutions would most benefit from, based on 60 post-course survey responses. Training workshops and courses were identified by 55 respondents (90.2 %), research collaboration by 55 (90.2 %), resident/fellow exchange programs by 54 (88.3 %), equipment donation by 48 (78.7 %), telemedicine consultation by 45 (73.8 %), and faculty exchange programs by 43 (70.5 %). These data demonstrate strong demand for sustained, multifaceted international partnerships beyond isolated training events.

Over half of participants (N = 35, 57.4 %) reported that their institutions already maintain partnerships aimed at supporting neurosurgical development. However, an overwhelming majority (N = 52, 85.2 %) expressed interest in participating in institutional twinning programs in the future. The types of support identified as most beneficial included training workshops and courses (N = 55, 90.2 %), research collaboration opportunities (N = 55, 90.2 %), and resident or fellow exchange programs (N = 54, 88.3 %) (Fig. 4). These data reveal strong demand for sustained international collaboration beyond isolated training events.

## 5 Discussion

### 5.1 Impact and significance of the inaugural course

The first Pan-African EANS-supported training course in neurosurgery achieved exceptional success, with participants reporting marked gains in knowledge and very high overall satisfaction. These improvements indicate a clinically meaningful educational impact and strong momentum for continued program development. While self-reported knowledge measures have inherent limitations, previous studies in similar contexts have demonstrated correlation between subjective knowledge gains and improved clinical confidence and competency (Waterkeyn et al., 2023).

This initiative represents the first comprehensive, continent-wide training program specifically adapted for African neurosurgeons practicing in LMIC settings. Unlike previous localized or subspecialty-specific efforts(Cadieux et al., 2025; Waterkeyn et al., 2023), this course establishes a scalable, reproducible model that can be systematically expanded across subspecialties and geographic regions. The Pan-African scope – with participation from 19 countries across all major African regions – demonstrates feasibility of large-scale collaborative educational initiatives in resource-limited settings.

### 5.2 Educational model effectiveness

Participants' strong preference for interactive formats – particularly discussion groups and breakout sessions – validates the EANS training course pedagogical approach and confirms its applicability in African contexts. These small-group, case-based learning formats received significantly higher ratings than traditional lectures, supporting educational theories emphasizing active learning and peer collaboration (Freeman et al., 2014). The innovative breakout session format, specifically developed for EANS training courses over decades of refinement, proved particularly effective when bridging knowledge gaps between different healthcare systems.

The high rating for content adaptation to LMIC settings confirms the strategic success of selecting faculty with global neurosurgery experience who could effectively tailor complex technical content to resource-limited practice realities. This contextualization is critical, as teaching advanced techniques without considering equipment availability, patient socioeconomic factors, and healthcare infrastructure limitations would diminish practical applicability.

It is worthy of notice that self-reported knowledge scores in this study primarily capture perceived competence and confidence rather than objective technical performance. Such measures are nonetheless relevant, as increased confidence and clearer conceptual understanding are important prerequisites for more appropriate case selection, safer intraoperative decision-making, and more timely referral once participants return to their home institutions. However, without objective testing or longitudinal follow-up, these data cannot demonstrate direct improvements in operative skill or patient outcomes, underscoring the need for future course iterations to incorporate formal assessments and longer-term evaluation of practice patterns.

### 5.3 Infrastructure challenges and training needs

Survey data highlight substantial infrastructure gaps among participating centers. Although aneurysm clipping was available in a majority of respondents’ institutions, endovascular capability and neuronavigation were reported by only a minority, and a small but important proportion lacked all major equipment categories surveyed. These findings confirm previous reports documenting persistent equipment shortages across African neurosurgical centers (Dewan et al., 2019; Meara et al., 2015). Because participants were mainly drawn from tertiary and university-affiliated referral hospitals, these data likely overestimate equipment availability compared with the broader African neurosurgical landscape, where previous capacity studies show far more limited access in decentralized, non-tertiary settings. This contrast between well-resourced referral centers (i.e., Muhimbili Orthopaedic and Neurosurgery Institute – MOI in Dar es Salaam, Tanzania), and under-resourced regional facilities underscores the pronounced heterogeneity of neurosurgical practice across the continent. While progress has been made in establishing basic neurosurgical services, most institutions remain unable to perform high-volume, advanced vascular or skull base procedures without substantial equipment investments.

Interestingly, despite limited access to advanced technology, the majority of participants came from institutions where skull base procedures are performed, suggesting that many African neurosurgeons are attempting complex cases with suboptimal resources. Yet, the high proportion of respondents should be interpreted in light of the broad operational definition used. In many participating centers, “skull base surgery” encompasses a wide spectrum ranging from relatively straightforward cranial base lesions to more complex cases, often performed without dedicated skull base units or advanced technology. Consequently, these data reflect the diversity of skull base pathology encountered and managed in African tertiary and referral institutions, rather than uniform access to highly specialized skull base programs across the continent. This reality underscores the critical importance of providing high-quality theoretical and technical training that can guide safe surgical decision-making even in resource-constrained environments.

### 5.4 Demand for hands-on training

Participant feedback consistently highlighted the need for more hands-on components, particularly cadaveric dissection, simulation, and live surgical observation, to complement the lecture and case-based curriculum. Hands-on exposure is especially critical in LMIC settings, where limited case volumes in some subspecialties and constrained access to advanced technology can delay the acquisition of complex technical skills; structured skills laboratories and simulation-based bootcamps provide a safe environment to practice key steps repeatedly before applying them in the operating room. Cadaveric and simulation-based neurosurgical training initiatives in LMIC regions – including regional skills laboratories in sub-Saharan Africa and low-cost simulation bootcamps – have demonstrated improvements in anatomical understanding, technical performance, and trainee confidence using affordable, context-adapted models (Winkler-Schwartz et al., 2019; Bakhshi et al., 2022; Konan et al., 2022; Torres et al., 2023).

Informed by these prior experiences, integrating structured hands-on modules into subsequent Pan-African courses – via partnerships with regional cadaver laboratories, development of low-cost simulators, and facilitated live-surgery observerships – will be a key priority to better translate theoretical learning into operative competence, especially in response to African trainees’ expressed needs. Embedding such intensive practical components within a large, continent-wide course is, however, logistically highly challenging given the high number of participants, but exploring scalable formats and satellite hands-on activities linked to the Pan-African course will be a central focus of future iterations.

### 5.5 Partnership and sustainability opportunities

The overwhelming interest in institutional twinning programs and the high demand for training collaborations, research partnerships, and exchange programs provides a clear roadmap for sustained GHC initiatives beyond isolated courses. Twinning partnerships between high-income country academic centers and LMIC neurosurgical departments have proven successful in other African contexts, demonstrating that long-term collaborative relationships can achieve sustainable capacity building (Waterkeyn et al., 2023; Haglund et al., 2017).

The willingness of the vast majority of participants to serve as future faculty and to host subsequent courses indicates strong potential for progressive Africanization of the program and the emerge of a self-sustaining continental educational network,

### 5.6 Scalability and expansion potential

This inaugural course establishes a validated template for expansion to additional subspecialties and geographic locations. Participants prioritized pediatric neurosurgery and neuro-oncology. The four-year EANS course cycle – covering vascular/skull base, spine, neuro-oncology/pediatric neurosurgery, and neurotrauma/functional neurosurgery/peripheral nerve – could be systematically adapted for Africa, providing comprehensive subspecialty training over multiple years.

Geographic expansion to West, Central, and North African host sites would enhance accessibility for trainees from those regions and distribute the educational benefit more equitably across the continent.

### 5.7 Study limitations

This study has several important limitations. First, knowledge assessment relied on participant self-reporting rather than objective testing through written examinations or practical assessments. While self-reported measures provide valuable data on perceived competency and confidence, they do not directly measure knowledge retention or clinical skill acquisition. Future iterations should incorporate objective knowledge assessments to more rigorously evaluate educational impact.

Second, findings represent a single course focused on two subspecialties at one geographic location and may not generalize to all neurosurgical topics or African regions. The specific needs and baseline knowledge levels may vary substantially across different countries and training contexts.

Third, this study captures only immediate post-course outcomes. Long-term follow-up is essential to determine whether reported knowledge gains translate into sustained knowledge retention, changes in clinical practice patterns, increased surgical volumes for complex procedures, improved patient outcomes, and enhanced career development for participants. Future studies should include longitudinal assessment at 6-month and 12-month intervals.

Fourth, the 59.8 % and 62.9 % survey response rates, while reasonable, introduce potential response bias. Participants who completed surveys may differ systematically from non-respondents in terms of engagement, satisfaction, or baseline characteristics.

Finally, resource constraints limited the ability to include extensive hands-on training components in the inaugural course. Future studies should evaluate the incremental benefit of adding simulation-based or cadaveric training modules.

### 5.8 Implications for global neurosurgical education

This initiative demonstrates that established training models developed in high-income countries can be successfully adapted and implemented in LMIC settings when contextualization, local partnership, and cultural sensitivity are prioritized. The collaborative approach – combining international both the African and the European faculty expertise with local leadership and strong support from continental and regional African surgical organizations – created legitimacy, contextual relevance, and sustainable infrastructure for program continuation.

The exceptional success of this inaugural course should encourage similar initiatives by other international neurosurgical societies and organizations. The model is highly scalable and could be replicated in other LMIC regions including South Asia, Southeast Asia, and Latin America, where similar training gaps exist.

## 6 Conclusions

The inaugural Pan-African EANS-supported training course in neurosurgery successfully established a viable, highly valued, and effective model for advanced neurosurgical education in Africa. This pioneering initiative represents the first comprehensive, continent-wide training program specifically designed for neurosurgeons practicing in low- and middle-income settings. The collaborative approach – strategically combining international African and European expertise with local leadership and strong endorsement from continental African surgical organizations – coupled with emphasis on interactive, contextually adapted educational content, resulted in substantial self-reported knowledge improvements and exceptional participant satisfaction.

This initiative addresses a critical training gap while simultaneously strengthening the professional network of African neurosurgeons and fostering opportunities for sustained international partnership. The universal interest in program continuation, remarkable willingness of participants to serve as future faculty, and strong demand for institutional twinning relationships provide compelling evidence supporting the program's long-term sustainability and expansion potential.

Continued support and systematic geographic and subspecialty expansion of this program are essential for building sustainable neurosurgical capacity across the African continent. Future efforts might incorporate hands-on training components, implement objective knowledge assessments, and establish longitudinal outcome measures to document long-term impact on clinical practice and patient care. This inaugural course demonstrates that collaborative, adapted educational models can effectively enhance neurosurgical expertise in resource-limited settings, offering a reproducible framework for global neurosurgical capacity building.

## Conflict of interest

The authors declare that they have no known competing financial interests or personal relationships that could have influenced the work reported in this paper.
